# Association of breastfeeding with mental disorders in mother and child: a systematic review and meta-analysis

**DOI:** 10.1186/s12916-023-03071-7

**Published:** 2023-10-16

**Authors:** Polina Bugaeva, Inna Arkusha, Rinat Bikaev, Igor Kamenskiy, Aleksandra Pokrovskaya, Yasmin El-Taravi, Valeria Caso, Alla Avedisova, Derek K. Chu, Jon Genuneit, Gabriel Torbahn, Timothy R. Nicholson, Dina Baimukhambetova, Aigun Mursalova, Anastasia Kolotilina, Svetlana Gadetskaya, Elena Kondrikova, Mikhail Zinchuk, Renat Akzhigitov, Robert J. Boyle, Alla Guekht, Daniel Munblit

**Affiliations:** 1 Charité – Universitätsmedizin Berlin, Einstein Center for Neurosciences, Berlin, Germany; 2 V. Serbsky Federal Medical Research Center for Psychiatry and Narcology of the Ministry of Health of the Russian Federation, Moscow, Russia; 3 Moscow Research and Clinical Centre for Neuropsychiatry, Moscow, Russia; 4 Moscow City Clinical Hospital After V.M. Buyanov, Moscow, Russia; 5 Department of Brain Sciences, Faculty of Medicine, Dementia Research Institute UK, Imperial College London, London, UK; 6 Endocrinology Research Centre, Moscow, Russia; 7 Stroke Unit, Santa Maria Della Misericordia Hospital, University of Perugia, Perugia, Italy; 8 Division of Clinical Immunology & Allergy, Department of Medicine, and Department of Health Research Methods, Evidence & Impact, McMaster University, Hamilton, Canada; 9 Department of Pediatrics, Pediatric Epidemiology, Medical Faculty, Leipzig University, Leipzig, Germany; 10 German Center for Child and Youth Health, Leipzig, Germany; 11 Department of Pediatrics, Paracelsus Medical University, Klinikum Nürnberg, Universitätsklinik Der Paracelsus Medizinischen Privatuniversität Nürnberg, Nuremberg, Germany; 12 Department of Pediatrics, Paracelsus Medical University, Salzburg, Austria; 13 Institute of Psychiatry, Psychology and Neuroscience, King’s College London, London, UK; 14 Department of Paediatrics and Paediatric Infectious Diseases, Institute of Child’s Health, I.M. Sechenov First Moscow State Medical University, Sechenov University, Moscow, Russia; 15 National Heart and Lung Institute, Imperial College London, London, UK; 16 Care for Long Term Conditions Division, Florence Nightingale Faculty of Nursing, Midwifery and Palliative Care, King’s College London, London, UK; 17 I.M. Sechenov First Moscow State Medical University, Sechenov University, Moscow, Russia; 18 Department of Infectious Disease, Faculty of Medicine, Imperial College London, London, United Kingdom

**Keywords:** Anxiety disorders, Breastfeeding, Child health, Depressive disorders, Maternal health, Mental health, Schizophrenia, Systematic review

## Abstract

**Background:**

Breastfeeding has long been associated with numerous benefits for both mothers and infants. While some observational studies have explored the relationship between breastfeeding and mental health outcomes in mothers and children, a systematic review of the available evidence is lacking. The purpose of this study is to systematically evaluate the association between breastfeeding and mental health disorders in mothers and children.

**Methods:**

We systematically searched MEDLINE and EMBASE from inception to June 2, 2023. The inclusion criteria consisted of all studies evaluating links between breastfeeding and development of mental health disorders in children and mothers. Risk of bias was assessed using the Newcastle–Ottawa Scale (NOS) while grading of Recommendations Assessment, Development and Evaluation (GRADE) was used to assess the certainty of evidence. A random-effects meta-analysis was used if possible, to estimate the odds ratio for the association between breastfeeding and mental health outcomes. The Mantel–Haenszel method was utilised for pooling ORs across studies. Study heterogeneity was assessed using the *I*^2^ statistic.

**Results:**

Our review identified twenty-one original study. Of these, 18 focused on the association between breastfeeding and child health, assessing depressive disorders, schizophrenia, anxiety disorders, eating disorders and borderline personality disorder. Three studies evaluated the associations between breastfeeding and maternal mental health disorders. Three studies looking at outcomes in children showed no significant association between breastfeeding and occurrence of schizophrenia later in life (OR 0.98; 95% CI 0.57–1.71; *I*^2^ = 29%). For depressive disorders (5 studies) and anxiety disorders (3 studies), we found conflicting evidence with some studies showing a small protective effect while others found no effect. The GRADE certainty for all these findings was very low due to multiple limitations. Three studies looking at association between breastfeeding and maternal mental health, were too heterogeneous to draw any firm conclusions.

**Conclusions:**

We found limited evidence to support a protective association between breastfeeding and the development of mental health disorders in children later in life. The data regarding the association between breastfeeding and maternal mental health beyond the postnatal period is also limited. The methodological limitations of the published literature prevent definitive conclusions, and further research is needed to better understand the relationship between breastfeeding and mental health in mothers and children.

**Supplementary Information:**

The online version contains supplementary material available at 10.1186/s12916-023-03071-7.

## Introduction

Mental health disorders continue to be a major global medical and societal burden, affecting individuals of all ages. These disorders are linked to significant morbidity, disability, and mortality rates [1, 2]. Prior to the COVID-19 pandemic, the Global Burden of Disease Study estimated that 264 million people worldwide suffered from depression, while bipolar disorder and schizophrenia affected 45 million and 20 million individuals respectively [3]. The COVID-19 pandemic additionally resulted in increase in prevalence of major depressive disorder and anxiety disorders [4]. Individuals residing in regions with political, social, and humanitarian problems are particularly susceptible to mental health disorders [2]. In developed countries, patients often face stigmatisation and neglect by society [2, 5, 6].

The aetiology and mechanisms of mental disorders are complex and not fully understood with multiple factors, hereditary, social and environmental, proposed among the main contributors to their development which limits potential for preventive measures [7]. Potential exposures during infancy and early childhood were extensively investigated and associations with mental health disorders development were reported [8].

Breastfeeding is associated with multiple beneficial effects for maternal and child health. Leading local and international organisations, such as the World Health Organization (WHO) [9], the European Commission for Public Health (ECPH) [10] and the American Academy of Pediatrics (AAP), produced guidelines recognising exclusive breastfeeding as an optimal feeding method during the first 6 months of life [11]. Breastfeeding is a well-established contributor both to child socioemotional and neurocognitive development [12, 13]. The reason commonly named behind this association is presence of potentially beneficial constituents, such as immunological biomarkers and long-chain polyunsaturated fatty acids (LC-PUFAs), that cannot be fully replaced by human milk substitutes [14–16], association between breastfeeding and attachment [17], skin-to-skin contact and socio-emotional aspects [14].

Although some attempts to investigate associations between breastfeeding and mental disorders were made, comprehensive assessment of available evidence is still lacking. Therefore, the aim of this systematic review is to comprehensively assess available up to date evidence on the associations between breastfeeding and development of mental health disorders in children and mothers to provide an impetus for further research in the field and improve our understanding of the topic.

## Methods

This systematic review is reported in accordance with the recommendations set forth by the Preferred Reporting Items for Systematic Reviews and Meta-Analyses (PRISMA) statement [18]. The review protocol was registered with the National Institute for Health Research’s PROSPERO a priori (PROSPERO 2019 CRD42019134214).

### Search and screening

Studies were identified through searches of two electronic databases (Medline and Embase via OVID) from inception to June 3, 2021, using both free text and medical subject headings (MeSH) terms. Additional search was performed on June 2, 2023, to screen for recent papers. The search strategies are presented in Additional file 1: Box S1. In addition, the publications cited in the reference lists of the included studies and previously published review articles were carefully screened to ensure that no original published data had been missed.

Pairs of authors (PB, IA, DB, AM and RB) independently conducted the title and abstract screening. Any disagreements between the screeners were resolved via consensus or a third reviewer (DM).

### Eligibility criteria

#### Types of studies

Any randomised controlled trials (RCTs), quasi-RCTs, as well as cohort (prospective or retrospective) studies, nested case–control studies, other case–control studies and cross-sectional studies (including those with retrospective data) were included.

#### Types of participants

No restrictions to specific population and high-risk groups (e.g. cohorts of patients with family history of mental illnesses) were applied.

#### Types of interventions or exposures

Type/duration of breastfeeding. Our systematic review encompassed all infants who were fed breast milk, irrespective of whether it was delivered directly through breastfeeding or administered via a bottle of expressed milk.

#### Comparator

The comparison was made with the individuals who were not breastfed (any other type of feeding) and/or breastfeeding of varying duration.

#### Outcomes of interest

The outcomes of interest are as follows: depressive disorders, bipolar disorder, anxiety disorders, phobia, social anxiety disorders, panic disorder, obsessive–compulsive disorder, separation anxiety disorder, personality disorders, neuroticism, feeding and eating disorders, anorexia, bulimia, binge eating disorder, posttraumatic stress disorder, dysphoria, dissociative disorders, schizophrenia, anaclitic depression, attachment, suicide, alcohol addiction. We did not apply any restrictions pertaining to the diagnosis of the condition.

This systematic review delved into the associations between breastfeeding and designated mental health outcomes, separately assessing these relationships for (a) children and (b) mothers.

Studies with follow-up period less than 12 months since birth were excluded. Manuscripts investigating associations between breastfeeding and autism spectrum disorders (ASD) and attention deficit hyperactivity disorders (ADHD) were excluded, as systematic reviews regarding these conditions were published recently [19, 20]. Premature infants are at an increased risk for chronic medical conditions and developmental outcomes, including mental health disorders [21]. However, the underlying factors that contribute to these risks may differ from those in full-term infants. Therefore, including studies that focus specifically on premature infants may introduce heterogeneity into the analysis, making it difficult to draw conclusions about the association between breastfeeding and mental health outcomes in a general population. To ensure the validity and generalisability of the results, we excluded research papers in which the subjects were premature infants from this systematic review. We also excluded studies providing no specific diagnosis in patients within the cohort of interest. As this topic has been addressed in a previous systematic review, studies examining the relationship between breastfeeding and postnatal maternal depression were excluded [22].

### Data extraction

Pairs of authors (PB, IA, DB, AM, AP and RB) independently conducted the data extraction. The extracted data included study design, country of the study, population characteristics, age of children or/and follow-up period, sample size and follow-up rate, number of cases and controls in exposed and non-exposed group, definition of exposure and outcome, methods of outcome assessment, effect estimates with 95% Cis and confounders included in the analysis.

### Data synthesis

All studies included in the systematic review were grouped by the outcome of interest and study type and then further grouped according to the type and/or duration of breastfeeding and effect measure (e.g. odds ratio, hazard ratio). Final groups with three or more studies with comparable exposure and outcome definition were considered suitable for meta-analysis. For outcomes where the original studies did not report both the number of participants in the exposed and non-exposed groups and the odds ratios, meta-analysis was not conducted. This is because such information is necessary to calculate the weights of the studies and estimate the overall effect size.

A random-effects meta-analysis was conducted to estimate the odds ratio (OR) and corresponding 95% confidence interval for the association between breastfeeding and mental health outcomes. The Mantel–Haenszel method was used to pool the Ors across studies. The results were considered statistically significant if the 95% CI did not include the null value of 1.0. Heterogeneity across studies was assessed using the *I*-squared (*I*^2^) statistic.

### Risk of bias assessment

Risk of bias assessment was performed by two authors (PB and RB or YET) independently using Newcastle–Ottawa Scale (NOS), which provides different assessment strategies for case–control and cohort studies [23]. Assessment according to NOS total score was reported as follows: very good = 9–10, good = 7–8, satisfactory = 5–6, unsatisfactory = 0–4. Selection of NOS instead of Risk Of Bias In Non-randomised Studies—of Interventions (ROBINS-I) was based on similar reliability but better applicability [24]. Results of NOS evaluation are outlined in Additional file 1: Tables S1–S3.

The grading of Recommendations, Assessment and Evaluation (GRADE) approach was used to evaluate the certainty of evidence for diseases of interest where pooled analyses and/or narrative synthesis was possible. As per suggested approach it was classified into high, moderate, low and very low [25]. Data from observational studies were considered low-quality evidence unless there was plausible evidence which would suggest upgrade of evidence certainty [26].

## Results

### Synthesis

**Fig. 1:**
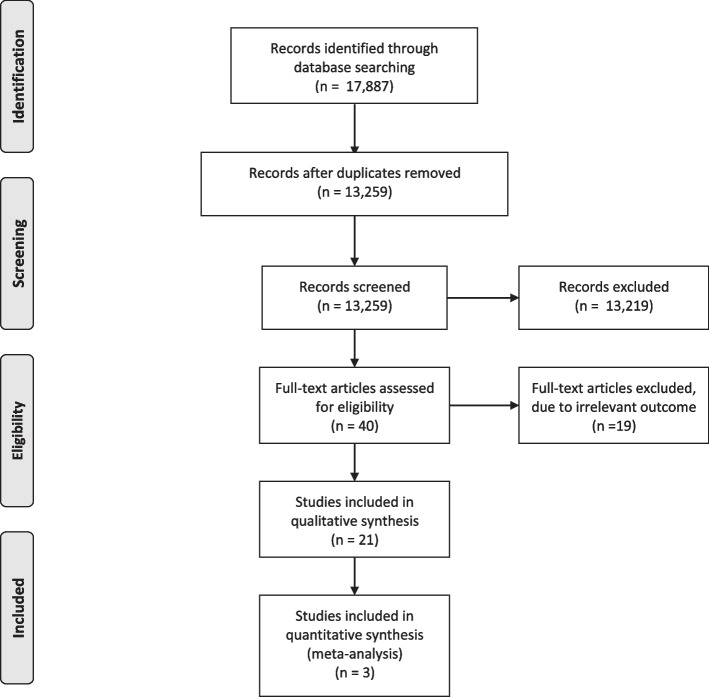
PRISMA flow diagram

A total of 17,887 items were identified through initial searches, and 40 articles met the inclusion criteria after duplicate removal and title/abstract screening (Fig. 1). Full-text manuscripts were assessed, and 19 articles were excluded, resulting in 21 articles included in the qualitative synthesis, with 18 investigating association between breastfeeding and child health and three looking at maternal health outcomes. Of these, three studies were included in the quantitative synthesis for the outcome of schizophrenia. The included studies were published between 1997 and 2023 and were all observational, comprising of 6 case–control studies [27–32], 5 retrospective studies [33–37] and 9 prospective cohort studies [38–46] and one cross-sectional study with retrospective assessment of the exposure [47].

The studies investigated associations between breastfeeding and mental health disorders in children and maternal mental health disorders. Specifically, eight studies assessed depressive disorders [33, 34, 36, 40, 41, 43, 45, 47], six investigated schizophrenia [28–30, 32, 37, 38] and five looked at anxiety disorders [31, 41, 43, 45, 47], while eating disorders [42] and borderline personality disorder [27] were assessed in one study each. In one study, the prevalence of mental health disorders in children was described without specifying the outcome [44].

Three studies evaluated the associations between breastfeeding and maternal mental health disorders [35, 39, 46].

### Participant characteristics

Studies were carried out in twelve different countries, with the majority of the research being conducted in the European and Australasian regions. There were three studies conducted in China [33, 34, 40], three in the USA [36, 39, 42], three in Australia [41, 43, 46], and in the United Kingdom [32, 37, 47], two in Brazil [44, 45] and one each in South Korea [35], Japan [29], South Africa[28], Italy [30], Turkey [31], Denmark [38] and Germany [27].

Sample size ranged from 160 [39] to 186,452 [25] participants in cohort studies and between 100 [28] and 450 [31] in case–control studies. Most of the studies followed children up to the adolescence with the maximum follow-up duration of 40 years.

### Breastfeeding definition and reporting

There was a substantial variation in breastfeeding reporting and definitions used. Some studies collected already predefined data from the registries [46], others used structured standardised (e.g. Pre-/Postnatal Stress Questionnaire (NPQ-PSQ) [27], Growing Up Today Study (GUTS) questionnaire [42]) and non-standardised questionnaires and interviews [28, 30, 41, 44, 45, 47, 31–34, 36, 37, 39, 40] obtaining the data prospectively or retrospectively. Exclusive breastfeeding was usually defined as breastfeeding without intake of “any other food”.

### Association between breastfeeding and mental health in children

#### Schizophrenia

Six studies investigated schizophrenia, with four using case–control design [28–30, 32], and two cohort studies, a prospective [38] and a retrospective [37] (Table 1). The outcome of interest was defined in accordance with Diagnostic and Statistical Manual of Mental Disorders (DSM)-III [32], DSM-IV [28–30], ICD 9 [37], International Classification of Diseases (ICD)-8 code 295 or ICD-10 code F20 [28]. In a single study, Sorensen and co-authors defined schizophrenia as bizarre delusions, delusions of control, abnormal affect, autism, hallucinations and disorganised thinking [38].

**Table 1:** Characteristics of the included studies investigating associations between breastfeeding and schizophrenia

Study	Country	Sample size (cases)	Outcome criteria	Age at outcome assessment, years	Exposure	Reference	Crude OR (95% CI)	Adjusted OR (95% CI)	Adjusted variables	NOS score^a^
Case–control studies										
Hartog 2007 [28]	South Africa	100 (50)	DSM-IV	21.7 ± 8.6	Ever	Never	0.58 (0.23–1.46)	NR	NA	6
Amore 2003 [30]	Italy	366 (113)	DSM-IV Axis I Disorders (SCID-I)	eBF ≥ 4 months 22.1 ± 6.3 Others 20.8 ± 4.9	Ever	Never	0.91 (0.43–1.94)	NR^b^	Age, sex, birth weight, severity of the disease, birth order	6
					eBF ≥ 4 months	eBF < 4 months	1.21 (0.69–2.13)			
					eBF ≥ 4 months	Never	0.99 (0.44–2.23)			
Sasaki 2000 [29]	Japan	300 (100)	DSM-IV	Cases: M: 49 ± 16 F: 32 ± 9 Controls: M: 50 ± 16 F: 31 ± 10	Ever	Never	1.59 (0.74–3.4)	NR	NA	6
					eBF ≥ 1 month	BF ≤ 1 month or never	0.9 (0.56–1.46)			
					eBF ≥ 1 month	Never	1.43 (0.65–3.15)			
					eBF ≥ 3 month	BF ≤ 3 months or never	1.13 (0.7–1.83)			
McCreadi 1997^e^ [37]	United Kingdom	137 (45)	ICD-9	NR	Ever	Never	0.66 (0.31–1.43)	NR	NR	7
Cohort studies										
Leask 2000 [32]	United Kingdom	NSHD: 4476 (29)	DSM-III	43.6	Ever	Never	0.97 (0.41–2.27)	NR	Gender and social class	8
					BF > 1 month	BF < 1 month	0.65 (0.24–1.78)	NR		
					BF > 1 month	Never	0.89 (0.37–2.16)	0.86 (0.36–2.1)		
					BF < 1 month	Never	1.36 (0.43–4.32)	1.35 (0.43–4.3)		
		NCDS: 14501 (29)	DSM-III	7, 11, 16, 23, 33^d^	Ever	Never	1.46 (0.62–3.42)	NR	Gender and social class	
					BF > 1 month	BF < 1 month	1 (0.42–2.4)	NR		
					BF > 1 month	Never	1.46 (0.59–3.62)	2.03 (0.73–5.7)		
					BF < 1 month	Never	1.46 (0.53–4.02)	1.99 (0.65–6.1)		
Sorensen 2005 [38]	Denmark	6841 (93)	ICD-8 code 295 or ICD-10 code F20	NR^c^	≤ 2 weeks	> 2 weeks	1.80 (1.18–2.75)	1.75 (1.14–2.68)	Gender, parental social status at 1 year of age	8
								1.73 (1.13–2.67)	Maternal schizophrenia, single mother status AND gender, parental social status at 1 year of age	

*F* female, *M* male, *NA* not applicable, *NCDS* National Child Development Study, *NSHD* National Survey of Health and Development (NSHD) cohort, *NR* not reported

^a^Newcastle Ottawa (NOS) Score: very good = 9–10, good = 7–8, satisfactory = 5–6, unsatisfactory = 0–4

^b^Authors reported that no significant associations were found in logistic regression analyses, but numerical results were not presented

^c^Participants were recruited between October 1959 and December 1961. Primary outcome was extracted from the Danish Psychiatric Central Register between 1 April 1969 and December 1999

^d^Patients discharged from psychiatric hospitals between 1974 and 1986 were identified from the Mental Health Enquiry

^e^McCreadi et al. used siblings as a control group

The study risk of bias determined by means of NOS was good in cohort studies, while case–control studies were generally of satisfactory risk of bias (range satisfactory to good). The common flaw was lack of adjustment for potentially significant confounders such as family history of schizophrenia, with four studies presenting crude data only [28–30, 37]. Exposure to breastfeeding was self-reported by parents, and for several case–control studies, the recall period exceeded 20 years [32, 37, 38].

All but one study [38] found no association between breastfeeding and schizophrenia, Sorensen et al. reported an association between breastfeeding for two weeks or less and increased risk of schizophrenia adjOR 1.73 (95% CI 1.13–2.67) upon adjustment for maternal schizophrenia, single mother status, sex and parental social status at 1 year of age.

**Fig. 2:**
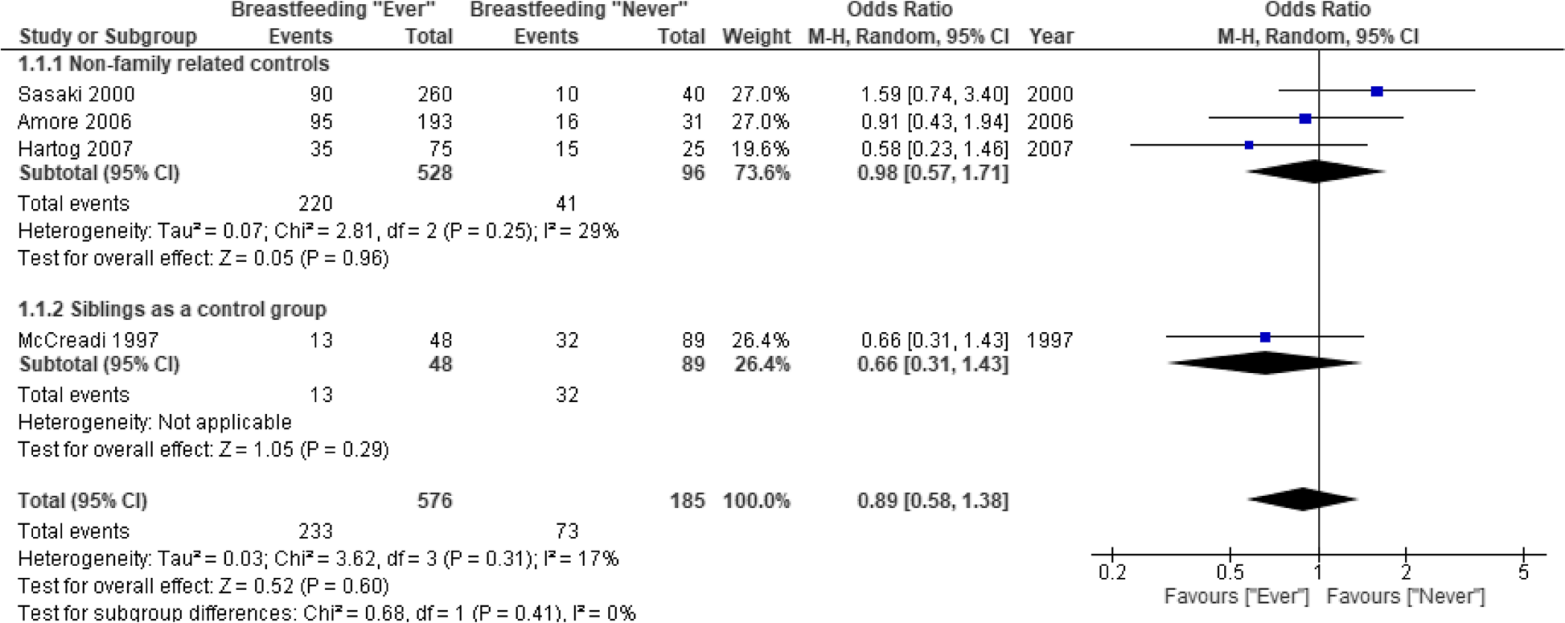
Meta-analysis of case–control studies. Breastfeeding (ever vs. never) and risk of schizophrenia. 1.1.1. Primary analysis, which includes studies using non-family related subjects as a control group. 1.1.2. Sensitivity analysis, which included McCreadi et al. study, which used siblings as a control group

Heterogeneity in methodology and lack of relevant outcome reporting did not allow for meta-analysis of the data from cohort studies. The pooled data from three case–control studies (*n* = 528) showed no significant association between breastfeeding and schizophrenia later in life OR 0.98 (95% CI 0.57–1.71) [28–30] (Fig. 2). In the sensitivity analysis, addition of another study [37] which used siblings as a control group did not change the results OR 0.89 (95% CI 0.58–1.38). The GRADE certainty of evidence was very low due to risk of bias and serious imprecision (Table 2).

**Table 2:** Exposure to breastfeeding compared to absence of breastfeeding in protection of children mental health. Grading of Recommendations Assessment, Development and Evaluation (GRADE) assessment

Certainty assessment							Summary of findings				
**Participants (studies)** **Follow-up**	**Risk of bias**	**Inconsistency**	**Indirectness**	**Imprecision**	**Publication bias**	**Overall certainty of evidence**	**Study event rates (%)**		**Relative effect (95% CI)**	**Anticipated absolute effects**	
							**With absence of breastfeeding**	**With exposure to breastfeeding**		**Risk with absence of breastfeeding**	**Risk difference with exposure to breastfeeding**
**Schizophrenia**											
3 observational studies^a^	Serious	Not serious	Not serious	Serious	Unclear	⨁○○○ Very low	261 cases 363 controls		**OR 0.98** (0.57 to 1.71)	**Satisfactory**	
										156 per 1,000	**3 fewer per 1000** (from 61 fewer to 84 more)
**Depressive disorders**											
5 observational studies^b^	Serious^c^	Serious^d^	Serious^e^	Not serious	Unclear	⨁○○○ Very low	There is conflicting evidence regarding associations between breastfeeding and depressive disorders development with some studies showing small protective effect and others reporting no effect				
**Anxiety disorders**											
3 observational studies	Serious^f^	Serious^g^	Not serious	Not serious	Unclear	⨁○○○ Very low	There is conflicting evidence regarding associations between breastfeeding and anxiety disorders development with one cohort study (*n* = 3657) reporting no effect while one cross-sectional study (*n* = 98,702) and one case–control study (*n* = 450) demonstrating protective effect				

*CI* confidence interval, *OR* odds ratio

^a^McCreadi et al. 1997 study used siblings as a control group. Outcomes of the sensitivity analysis, which included this study did not differ (OR 0.89 (95% CI 0.58–1.38)) from the primary analysis. Two cohort studies provided conflicting evidence with Sorensen et al. 2005 demonstrating reduced risk and Leask et al. 2000 no effect

^b^De Mola et al. 2016 study compared different durations of breastfeeding but did not provide “ever” vs. “never” comparison

^c^Three studies were ranked as “satisfactory” using NOS score, while one was rated as “good”

^d^The direction and magnitude of effect varied across the studies. Overall, the results showed either small protective effect or no association between breastfeeding and depressive disorders

^e^We judged the evidence to have potential serious indirectness due to substantial variability in the outcome measure used

^f^One of two studies was rated as “unsatisfactory” as per NOS

^g^The direction and magnitude of effect varied across the studies

#### Depressive disorders

Five studies (three retrospective [33, 34, 36], two prospective cohorts [31, 36] and one cross-sectional study with retrospective assessment of the exposure which derived data from the UK biobank cohort [47]) investigated depressive disorders (Table 3).

**Table 3:** Characteristics of the included studies investigating associations between breastfeeding and depressive disorders

Study^c^	Country	Sample size (cases)	Outcome criteria	Age at outcome assessment, years	Exposure	Reference	Crude OR (95% CI)	Adjusted OR (95% CI)	Adjusted variables	NOS score^a^
Cohort studies										
Zhong 2013 [33]	China	3582 (86)	MINI-KID 5.0	6–14	Never^b^	Ever^b^	2.07 (1.39–3.09)	1.88 (1.28–2.49)	Age, school attendance, parental marital status, family type, dominant caregivers	6
De Mola 2016 [45]	Brazil	3542 (272)	MINI-KID 5.0	30	1–2.9 months	< 1 month	0.71 (0.51–0.97)	0.79 (0.53–1.16)	Sex, ancestry, birth weight, maternal age, maternal education, marital status, previous gestations, income at birth, smoking during pregnancy, type of delivery, assets index, mother nerve problems, father living together and father history of psychiatric illness	4
					3–5.9 months	< 1 month	0.78 (0.57–1.08)	0.93 (0.63–1.38)		
					≥ 6 months	< 1 month	0.69 (0.51–0.95)	0.77 (0.53–1.12)		
Allen 1998 [36]	USA	579 (141)	K-SADS	16.4 ± 1.2	Never	Ever	1.66 (1.13–2.45)	A: 1.68 (1.13–2.47); B: 1.71 (1.14–2.56); C: 1.64 (1.07–2.51)	MA: prenatal variables that were significantly associated with adolescent psychiatric disorder; MB: maternal depression, family cohesion, family conflict, adolescent physical symptoms; MC: other adolescent psychiatric disorders	5
Kwok 2013 [40]	China	5797 (261)	PHQ-9	11	Never	Mixed BF or EBF^b^ < 3 months	NR	1.14 (0.87–1.49)	Sex, age, survey mode, SEP (parents’ education, parents’ occupation, household income), mother’s birthplace, birth weight-for-gestational age z-score, birth order, second hand smoke exposure and parents’ age at birth	7
					Never	EBF ≥ 3 months	NR	0.77 (0.43–1.40)		
Huang 2019 [34]	China	1979 (227)	CBCL	6–11	< 6 months	Never	NR	0.79 (0.37–1.67)	Educational attainment of mothers, maternal medicine use during pregnancy, parents upbringing style, maternal alcohol drinking during pregnancy	6
					≥ 6 months	Never	NR	0.45 (0.23–0.91)		
Cross-sectional										
Liu 2023 [47]	United Kingdom	110,948 (53,690)	PHQ-9	> 50	Ever	Never	0.73 (0.71–0.75)	MSDP 0.86 (0.82–0.91) NMSDP 0.82 (0.79–0.84)	Sex, age, 10 principle components of population structure, smoking, alcohol use, and Townsend deprivation index	6

*K-SADS* Schedule for Affective Disorders and Schizophrenia for School-Age Children, *MINI-KID* Mini International Neuropsychiatric Interview for Children and Adolescents, *MSDP* maternal smoking during pregnancy, *NMSDP* no maternal smoking during pregnancy, *NR* not reported

^a^Newcastle Ottawa (NOS) score: very good = 9–10, good = 7–8, satisfactory = 5–6, unsatisfactory = 0–4

^b^Authors defined breastfeeding exposure as whether child was breastfed before the age of 1 year or not

^c^Hayatbakhsh et al. (*n* = 4502, NOS = 6) did not report odds/risk ratios, providing comparisons of continuous variables within each subscale of The Youth Self Report (YSR) of the Child Behavior Check List (CBCL) only. Authors reported statistically significant association between breastfeeding and “Anxiety/depression” domain for ≥ 4 months vs. “never” regression coefficient (95% CI) − 0.43 (− 0.78, − 0.09) and − 0.36 (− 0.70, − 0.01), respectively in adjusted models; Oddy et al. (*n* = 2366, NOS = 8) used a composite outcome “internalising complaints” that includes withdrawn, somatic complaints, anxiety, and depression. BF < 6 months vs. ≥ 6 months OR 1.21 (95% CI 1.0–1.46)

A wide range of instruments was used across the studies for outcome assessment: Mini International Neuropsychiatric Interview for Children and Adolescents (MINI-KID) 5.0 [33, 45], Child Behaviour Check List (CBCL) which is very consistent with DSM-V diagnostic categories [34]. Other scales included PHQ-9 (self-reported Patient Health Questionnaire-9) that is consistent with DSM-IV [40, 47] and Schedule for Affective Disorders and Schizophrenia for School-Age Children (K-SADS) which is compatible with DSM-III [36]. According to PHQ-9, depression was defined as having a total score of 11 or more mapping on to DSM-IV [40]. De Mola et al. used Beck’s Depression Inventory (BDI-II) validated for Brazilian population to assess severity of depressive symptoms [45].

The study risk of bias was satisfactory on average, ranging between 4 and 7 as per NOS.

Kwok et al. and De Mola et al. reported no association between breastfeeding and depressive disorders development [40, 45] upon adjustment for multiple potential confounders. Other two studies showed that absence of any breastfeeding was associated with an increased risk (adjOR 1.88 (95% CI 1.28–2.49 and adjOR 1.71 (95% CI 1.14–2.56) respectively) of depressive disorders later on in life (6–16 years of age) [33, 36]. Huang et al. reported a protective effect of breastfeeding only in children who were breastfed longer than 6 months compared to those who have never been breastfed adjOR 0.45 (95% CI 0.23–0.91), while shorter duration of breastfeeding was not associated with any protective effect adjOR 0.79 (95% CI 0.37–1.67) [34]. A very recent cross-sectional study from Liu et al., based on the data from the UK Biobank cohort suggested protective effect of breastfeeding in mothers smoking adjOR 0.86 (95% CI 0.82–0.91) and not smoking adjOR 0.82 (95% CI 0.79–0.84) during pregnancy [47]. The overall GRADE certainty of evidence was very low due to potential risk of bias, serious inconsistency and indirectness (Table 2).

There was a lack of studies reporting the number of participants in the exposed and non-exposed groups to conduct meta-analysis.

#### Anxiety disorders

Three studies (one case–control [31], one prospective cohort study [36] and one cross-sectional study with retrospective assessment of the exposure using the data from the UK biobank cohort [47]) investigated association between breastfeeding and anxiety disorders [22, 36] (Table 4).

**Table 4:** Characteristics of the included studies investigating associations between breastfeeding and anxiety disorders

Study^b^	Country	Sample size (cases)	Outcome criteria	Age at outcome assessment, years	Exposure	Reference	Crude OR (95% CI)	Adjusted OR (95% CI)	Adjusted variables	NOS score^a^
Cohort studies										
De Mola 2016 [45]	Brazil	3657 (438; 127)	MINI GAD	30	1–2.9 months	< 1 month	0.88 (0.69–1.13)	0.86 (0.64–1.16)	Sex, ancestry, birth weight, maternal age, maternal education, marital status, previous gestations, income at birth, smoking during pregnancy, type of delivery, assets index, mother nerve problems, father living together and father history of psychiatric illness	4
					3–5.9 months	< 1 month	0.91 (0.71–1.18)	1.02 (0.77–1.37)		
					≥ 6 months	< 1 month	0.81 (0.63–1.03)	0.79 (0.59–1.06)		
			SAD		1–2.9 months	< 1 month	0.72 (0.44–1.17)	0.61 (0.35–1.08)		
					3–5.9 months	< 1 month	0.79 (0.49–1.29)	0.90 (0.52–1.55)		
					≥ 6 months	< 1 month	0.75 (0.48–1.20)	0.66 (0.38–1.15)		
Case–control/cross-sectional										
Orengul 2018 [31]	Turkey	450 (195)	K-SADS	Cases: 11.50 ± 2.50; controls: ± 11.27–2.33	Ever	Never	0.17 (0.05–0.60)	NR	NR	8
Liu 2023 [47]	United Kingdom	98,702 (19,729)	GAD-7	> 50	Ever	Never	0.77 (0.75–0.80)	MSDP 0.87 (0.82–0.93) NMSDP 0.83 (0.79–0.87)	Sex, age, 10 principle components of population structure, smoking, alcohol use, and Townsend deprivation index	6

*K-SADS* Schedule for Affective Disorders and Schizophrenia for School-Age Children, *MINI* Mini International Neuropsychiatric Interview for Children and Adolescents, *MSDP* maternal smoking during pregnancy, *NMSDP* no maternal smoking during pregnancy, *NR* not reported

^a^Newcastle Ottawa (NOS) Score: very good = 9–10, good = 7–8, satisfactory = 5–6, unsatisfactory = 0–4

^b^Hayatbakhsh et al. (*n* = 4502, NOS = 6) did not report odds/risk ratios, providing comparisons of continuous variables within each subscale of The Youth Self Report (YSR) of the Child Behavior Check List (CBCL) only. Authors reported statistically significant association between breastfeeding and “Anxiety/depression” domain for ≥ 4 months vs. “never” regression coefficient (95% CI) − 0.43 (− 0.78, − 0.09) and − 0.36 (− 0.70, − 0.01), respectively in adjusted models; Oddy et al. (*n* = 2366, NOS = 8) used a composite outcome “internalising complaints” that includes withdrawn, somatic complaints, anxiety, and depression. BF < 6 months vs. ≥ 6 months OR 1.21 (95% CI 1.0–1.46)

For the primary outcome assessment, Orengul et al. investigated social anxiety disorder, unspecified anxiety disorders, generalised anxiety disorder, specific phobias, separation anxiety disorder and panic disorder using Schedule for affective disorders and schizophrenia for school-age children, present version (K-SADS-P), and the Revised Child Anxiety and Depression Scale (RCADS), total anxiety subscale [31], while De Mola et al. studied generalised anxiety disorder and social anxiety disorder using Mini International Neuropsychiatric Interview version 5.0 validated for Brazil [45].

The risk of bias of the studies varied from unsatisfactory to good.

De Mola and co-authors in their cohort study found no association between breastfeeding or its’ duration and anxiety disorders development [45], while a case–control study from Orengul et al. [31] found reduced risk in breastfed children when compared with those who have never been exposed to breast milk OR 0.17 (95% CI 0.05–0.60). Liu et al. found that breastfeeding in mothers smoking adjOR 0.87 (95% CI 0.79–0.87) and not smoking adjOR 0.83 (95% CI 0.79–0.87) during pregnancy being associated with less anxiety in a cross-sectional study based on the data from the UK Biobank cohort [47]. The overall GRADE certainty of evidence was very low due to potential risk of bias and serious inconsistency (Table 2).

There was a lack of studies reporting the number of participants in the exposed and non-exposed groups to conduct meta-analysis.

#### Depressive/anxiety disorders as a composite outcome

Two cohort studies assessed anxiety and depression as a composite outcome. Hayatbakhsh and co-authors used the Youth Self Report (YSR) of the Child Behaviour Check List (CBCL) which has the same format as the CBCL but with questions paraphrased in the first person [41]. Oddy et al. reported composite outcome “internalising complaints” that included withdrawn, somatic complaints, anxiety, and depression [43]. Hayatbakhsh et al. found breastfeeding for at least 4 months to be associated with lower scores of CBCL “anxiety/depression” domain at 14 years of age, while Oddy et al. reported breastfeeding for less than 6 months being associated with higher risk of internalising complaints OR 1.21 (95% CI 1.0–1.46).

There was a lack of studies reporting the number of participants in the exposed and non-exposed groups to conduct meta-analysis.

#### Other mental health disorders

Other conditions investigated in the reviewed manuscripts included borderline personality disorder (BPD) [27] and eating disorders [42] (Table 5).

**Table 5:** Characteristics of the included studies investigating associations between breastfeeding and other psychiatric disorders

Study	Country	Sample size (cases)	Outcome criteria	Age at outcome assessment, years	Exposure	Reference	Crude OR (95% CI)	Adjusted OR (95% CI)	Adjusted variables	NOS score^a^
Eating disorders										
Iron-Segev 2013 [42]	USA	6251 (1152)	Bulimic behaviours (Female)^b^	9 − 14	< 4 months	> 9 months	0,9 (0.8–1.1)	0,9 (0.8–1.1)	Gestational age/birthweight, age, age at menarche, maternal eating disorder diagnosis, and maternal pre-pregnancy body mass index, adolescent BMI, high weight concern scale and frequent dieting	4
					4 − 9 months	> 9 months	1.0 (0.9–1.2)	1.0 (0.9–1.2)		
		5542 (168)	Bulimic behaviours (Male)^b^		< 4 months	> 9 months	1.6 (1.1–2.3)	1.5 (1.0–2.3)		
					4 − 9 months	> 9 months	1.1 (0.8–1.7)	1.2 (0.8–1.7)		
		6436 (565)	Purging (F)^c^		< 4 months	> 9 months	1.1 (0.9–1.3)	1.1 (0.9–1.3)		
					4 − 9 months	> 9 months	1.0 (0.8–1.2)	1.0 (0.8–1.2)		
		6281 (881)	Binge eating (F)^d^		< 4 months	> 9 months	0.8 (0.7–1.0)	0.8 (0.7–1.0)		
					4 − 9 months	> 9 months	1.0 (0.8–1.2)	1.0 (0.8–1.2)		
		5691 (130)	Binge eating (M)^d^		< 4 months	> 9 months	1.3 (0.9–2.1)	1.3 (0.8–2.1)		
					4 − 9 months	> 9 months	1.0 (0.7–1.6)	1.0 (0.6–1.6)		
		5955 (307)	Self-reported eating disorder (F)		< 4 months	> 9 months	0.9 (0.7–1.2)	0.8 (0.6–1.1)		
					4 − 9 months	> 9 months	1.0 (0.8–1.3)	1.0 (0.8–1.3)		
Borderline personality disorder										
Schwarze 2015 [27]	Germany	200 (100)	DSM-IV	31.63 (cases), 32.02 (controls)	Never	Ever	3.32 (1.74, 6.34)	4.68 (1.88, 11.66)	Childhood trauma (CTQ total score), parental monthly income, maternal education, maternal age at child’s birth, maternal mental disorders, unwanted pregnancy, maternal age at birth, length of gestation, birth risk factors, birth complications and subject’s birth weight and size at birth	7

*F* female, *M* male, *NA* not applicable, *NCDS* National Child Development Study, *NSHD* National Survey of Health and Development (NSHD) cohort, *NR* not reported, *K-SADS* Schedule for Affective Disorders and Schizophrenia for School-Age Children, *MINI-KID* Mini International Neuropsychiatric Interview for Children and Adolescents

^a^Newcastle Ottawa (NOS) Score: very good = 9 − 10, good = 7 − 8, satisfactory = 5 − 6, unsatisfactory = 0 − 4

^b^Bulimic behaviours were defined as purging or binge eating at least monthly in the past year

^c^Purging was defined as using laxatives or force vomiting to lose weight or keep from gaining weight more than one time a month

^d^Binge eating was defined as eating binge at least once a month and feeling out of control while doing so

In a case–control study, Schwarze et al. investigated BPD defined as a pervasive pattern of impulsivity, emotional instability, identity disturbance and dysfunctional interpersonal relationships and diagnosed according to DSM-IV criteria for BPD [27]. Authors reported increased odds of BPD in those who have never been breastfed adjOR 4.68 (95% CI 1.88–11.66).

In a cohort study, Iron-Segev et al. assessed a broad range of eating disorders, including bulimic behaviours like purging, binge eating and other self-reported eating disorders like anorexia nervosa and bulimia nervosa. Purging was defined as using laxatives or force vomiting to lose weight or keep from gaining weight more than one time a month. The patient was considered as binge eater if eating binge at least once a month and feeling out of control while doing so was reported [42]. The study did not find any associations between breastfeeding duration (< 4 months, 4–9 months, > 9 months) and any eating disorder.

There was a lack of studies reporting the number of participants in the exposed and non-exposed groups to conduct meta-analysis.

### Association between breastfeeding and maternal mental health

Three studies (a retrospective [35] and two prospective cohorts [39, 46]) investigated associations between breastfeeding and long-term development of maternal mental health outcomes post lactation (Table 6). Among them, one looked at a variety of mental disorders, including schizophrenia, unipolar depression, bipolar affective disorder and anxiety disorders [46] and two assessed associations with maternal depression [35, 39].

**Table 6:** Characteristics of the included studies investigating associations between breastfeeding and maternal mental health outcomes

Study	Country	Sample size (cases)	Outcome criteria	Time/age at outcome assessment	Exposure	Reference	Crude OR/RR (95% CI)	Adjusted OR (95% CI)	Adjusted variables	NOS score^a^
Cohort studies										
Hahn-Holbrook 2013 [39]	USA	160 (24)	EPDS	up to 24 months after delivery	≥ 3 months	< 3 months	NR	NR	Maternal age, income, education, marital status, parity, preterm birth, maternal employment, ethnicity, and social support	7
Xu 2014^b^ [46]	Australia	186,452 (2940)	ICD-10 for schizophrenia	12 months after delivery	Never	BF	3.7 (2.5–5.6)	2.0 (1.3–3.1)	Maternal age, parity, maternal smoking status, socioeconomic status, maternal country of birth, delivery method, gestational age, admission to special care nursery or neonatal intensive care unit, and history of hospital admissions for mental illness	7
					Mixed	BF	2.0 (1.1–4.0)	1.7 (0.9–3.4)		
			ICD-10 for unipolar depression		Never	BF	1.0 (0.8–1.2)	1.0 (0.8–1.2)		
					Mixed	BF	1.0 (0.8–1.3)	1.0 (0.8–1.3)		
			ICD-10 for bipolar affective disorder		Never	BF	2.5 (1.4–4.3)	1.9 (1.1–3.5)		
					Mixed	BF	2.0 (0.9–4.5)	2.2 (1.0–5)		
			ICD-10 for anxiety disorders		Never	BF	0.5 (0.4–0.7)	0.6 (0.5–0.9)		
					Mixed	BF	1 (0.7–1.3)	0.9 (0.7–1.3)		
Park 2018 [35]	South Korea	1372 (139)	PHQ-9	> = 50 years of age	> 47 months	< 23 months	NR	0.33 (0.16 − 0.68)	Age, smoking, alcohol drinking, job status, education level, household income, marital status, number of pregnancies, obesity, diabetes mellitus, and hypertension	5
					24–46 months	< 23 months		0.61 (0.31–1.21)		

Hayatbakhsh et al. did not report odds/risk ratios, providing comparisons of continuous variables within each subscale of The Youth Self Report (YSR) of the Child Behavior Check List (CBCL) only

*F* female, *M* male, *NA* not applicable, *NCDS* National Child Development Study, *NSHD* National Survey of Health and Development (NSHD) cohort, *NR* not reported, *K-SADS* Schedule for Affective Disorders and Schizophrenia for School-Age Children, *MINI-KID* Mini International Neuropsychiatric Interview for Children and Adolescents

^a^Newcastle Ottawa (NOS) score: very good = 9 − 10, good = 7 − 8, satisfactory = 5 − 6, unsatisfactory = 0 − 4

^b^In the Xu et al. study, mixed feeding was defined as breastfeeding and infant formula. Exposure variable was assessed at the time of the discharge from hospital or discharge from care for home births

For depression assessment, instruments used included Patient Health Questionnaire 9 (PHQ-9), with scores of 10 or higher indicating depression [35], Edinburgh Postnatal Depression Scale (EPDS), with a cut-off of 10 scores being considered a minor depression [39] and ICD-10 codes at admission for each diagnosis of interest [46].

The study risk of bias determined by means of NOS varied from satisfactory to good.

Xu et. al reported association between absence of breastfeeding at the time of hospital discharge and higher risk of schizophrenia adjOR 2.0 (95% CI 1.3–3.1) and bipolar affective disorder adjOR 1.9 (95% CI 1.1–3.5) 12 months after delivery but found no protective effect against anxiety disorders and unipolar depression [46].

Park et al. reported protective effect of prolonged (> 47 months) breastfeeding against maternal depression in the postmenopausal period adjOR 0.33 (95% CI 0.16–0.68) [35].

Hahn-Holbrook and co-authors suggested that women who breastfed more frequently at 3 months postpartum showed greater subsequent declines in depressive symptomatology over time compared to women who breastfed less frequently and lower absolute levels of depressive symptoms by 24 months since birth [39].

Heterogeneity in outcome assessment did not allow for meta-analysis.

## Discussion

Breastfeeding has been shown to have a number of benefits for both mother and child. This systematic review assessed up-to-date evidence regarding breastfeeding association with mental health disorders in mother and child. Overall, the current evidence suggests that there is very weak or no association between breastfeeding and the development of mental health disorders. This conclusion is supported by the findings from several studies, including three studies that reported no association between breastfeeding and schizophrenia development in later life (very low evidence). There is conflicting evidence when it comes to associations between breastfeeding and the development of depressive and anxiety disorders, with some studies showing a small protective effect and others reporting no effect. Published literature on this topic has substantial methodological limitations that make it difficult to draw firm conclusions. As it is very hard to randomise breastfeeding exposure, particularly from the ethical perspective, all available evidence comes from observational research only. The GRADE assessment we provide serves a reflection of this limitation and possible biases.

In the published literature, several studies have examined the potential associations between breastfeeding and mental health disorders in both children and mothers. Among studies looking at outcomes in offspring, the majority assessed depressive disorders, schizophrenia and anxiety disorders. Single studies evaluated eating and borderline personality disorders. A small number of studies evaluated associations between breastfeeding and maternal mental health disorders, such as schizophrenia, bipolar affective disorder, anxiety disorders and depression.

### Association between breastfeeding and mental health in children

Schizophrenia was the outcome most often reported, with six studies investigating the potential association between breastfeeding and this disease using a variety of methodologies including case–control and cohort designs. The risk of bias of these studies was generally good for the cohort studies and satisfactory to good for the case–control studies, although several studies did not adequately adjust for potentially significant confounders such as family history of schizophrenia. Exposure to breastfeeding was self-reported by parents in most of these studies, and the recall period in some of the case–control studies exceeded 20 years. Overall, most of the studies found no significant association between breastfeeding and schizophrenia. Our meta-analyses of case–control studies demonstrated null effect. However, it is worth noting that a major limitation of case–control studies was lack of adjustment for confounding factors. Due to a small number of cohort studies reporting breastfeeding and schizophrenia, and heterogeneity in outcome reporting, we were unable to perform a meta-analysis. The only study reporting protective effect of breastfeeding for more than two weeks was the Copenhagen Perinatal Cohort [38].

Five cohort studies have investigated the potential association between breastfeeding and the development of depressive disorders in children. The risk of bias of these studies was satisfactory on average, according to the NOS. Two large cohort studies reported no effect, upon adjustment for multiple potential confounders. Two other studies found that an absence of exposure to breastfeeding was associated with an increased risk of developing depressive disorders later in life [33, 36]. The fifth study reported a protective effect of breastfeeding on the development of depressive disorders, but only in children who were breastfed for longer than 6 months, while shorter duration of breastfeeding was not associated with any protective effect [34]. We noted a substantial heterogeneity in approaches to the data collection, differing confounding factors used for adjustment and the outcome assessment with a variety of instruments used which may partially explain contrasting results. A large cross-sectional study with retrospective assessment of the exposure using the data from the UK biobank cohort found that breastfeeding is associated with a lower risk of depression development later in life. The major limitation of this study was related to collection of the data on breastfeeding with participants been asked of whether they were breastfed in their infancy [47]. Such data, based on individuals’ memories in their adulthood, is associated with a substantial risk of recall bias.

Three studies of varied methodologies and potential bias investigated the relationship between breastfeeding and anxiety disorders. The studies used different assessment tools and focused on various forms of anxiety disorders. De Mola et al. cohort study found no correlation between breastfeeding (or its duration) and the development of anxiety disorders [45]. In contrast, the case–control study by Orengul et al. found a decreased risk of anxiety disorders in children who were breastfed as opposed to those who were not [31]. In another cross-sectional study using data from the UK Biobank cohort, Liu et al. discovered an association between breastfeeding and reduced anxiety, irrespective of whether the mothers smoked during pregnancy or not [47], but study design was prone to substantial risk of bias described in a previous paragraph.

Very limited data are available regarding breastfeeding associations with eating disorders and borderline personality disorders. Iron-Segev and co-authors analysed data from the large prospective cohort study of children who are the offspring of female registered nurses participating in the Nurses’ Health Study II (NHS II) and found no associations between breastfeeding and eating disorders. Participant enrolment in the study at the child’s age of at least 9 years, lack of the data regarding infancy and childhood factors are among the primary limitations of this study. The selected sample represents nurses, predominantly white, from a middle and high socioeconomic status, which limit generalisability of the results. One small case–control study showed association between lack of breastfeeding exposure and development of borderline personality disorder development later in life. Reported confidence intervals were very wide and reflect small sample size which restricts extrapolation of these results to other populations.

### Association between breastfeeding and maternal mental health

A recent systematic review found an association between breastfeeding and a reduced risk of postpartum depression [22], but it is unclear if this effect persists beyond the first few months of a child’s life. Our review identified only three studies that examined the long-term effects of breastfeeding on maternal mental health. These studies were all cohort studies and used different methods to assess breastfeeding exposure and examined a range of mental health disorders. One large cohort study from Australia found that never breastfeeding was associated with a higher risk of hospitalisation for schizophrenia, bipolar affective disorders and substance-induced mental illness in the first year postpartum compared to women who breastfed their children [46]. Two other studies focused on breastfeeding duration. A small study from the USA found that women who breastfed more frequently at 3 months postpartum experienced a greater decline in depressive symptoms over time [39]. However, it is unclear if the sample size was sufficient for the analyses used and the study had limitations, including the use of self-report measures and a predominantly white, upper-middle class and married sample. A study in South Korea found that a longer cumulative duration of breastfeeding was associated with a decreased risk of postmenopausal depression [35]. However, this study also had limitations, including self-report measures. There is an apparent lack of understanding about the potential association between breastfeeding and long-term maternal mental health. There is a need for more high-quality research to examine the potential association between breastfeeding and long-term maternal mental health. This research should use rigorous methods to assess breastfeeding exposure and control for potential confounders, as well as examine a range of mental health outcomes. Additionally, research should consider the potential moderating factors that may influence the relationship between breastfeeding and maternal mental health, such as individual characteristics, social support and parenting stress.

The GRADE certainty of evidence for all the studied outcomes was very low. This means that there is a high degree of uncertainty about the results, and more high-quality research is needed to confirm these findings. Primary factors that can contribute to a very low GRADE certainty of evidence include risk of bias, serious imprecision, serious inconsistency and indirectness. The very low GRADE certainty suggests that there is a high degree of uncertainty about the results, and more research is needed to confirm these findings.

### Limitations

While the current evidence suggests that there is very weak or no association between breastfeeding and the development of mental health disorders, the review has several limitations that should be considered. First, there is a limited availability of high-quality studies that can provide conclusive evidence of the association between breastfeeding and mental health disorders. The current evidence base is primarily based on observational studies, which are subject to a higher risk of bias and confounding factors than randomised controlled trials. Additionally, there is heterogeneity in the study designs and outcomes, making it difficult to compare results across studies. Second, the studies included in this review may be subject to potential biases and confounding factors, such as recall bias or unmeasured confounding variables that were not accounted for in the studies. The studies may also have limitations in the way they assessed and reported breastfeeding exposure. It is also worth noting that the studies included may not be representative of all populations or settings, as the majority of the studies included predominantly white, middle and high socio-economic status individuals. Third, there is a lack of consistent evidence for some mental health outcomes, such as eating disorders and borderline personality disorder. There is also limited evidence available regarding the long-term effects of breastfeeding on maternal mental health. The GRADE certainty of evidence for all the studied outcomes was very low, indicating a high degree of uncertainty about the results and a need for more high-quality research to confirm these findings. Fourth, potential gaps may be associated with our exclusive focus on Medline and Embase databases, without incorporating insights from other relevant databases such as Scopus, CINHAL and PsycINFO, which might offer a broader perspective from allied health and social sciences. Abovementioned limitations should be carefully considered when interpreting the results presented in this manuscript.

### Potential directions for future research

Future work in the field may focus on the standardisation of definitions of breastfeeding and the use of consistent, validated tools for the assessment of mental health outcomes. Ideally, future research should consider prioritising cohort studies with larger sample sizes and longer, more regular, follow-up periods, to better understand the long-term implications of breastfeeding on mental health. As most studies lack comprehensive data pertaining to infant exposure to colostrum, and the definition of breastfeeding is frequently imprecise or unclear, there is an area for improvement. Consideration of potential confounding factors, such as family history of mental health disorders, is also crucial to ensure the validity of the findings, as lack of control for confounders was apparent in some of the published research.

Further research is particularly needed in relation to less frequently investigated mental health disorders, as well as in relation to maternal mental health outcomes. There is also a need for more diverse research populations, as most of existing studies were conducted in European and Australasian regions, potentially limiting the generalisability of the findings. Although very costly, development of international consortia focused on prospective, register-oriented data collection could improve the knowledge in the field. A policy-driven, comprehensive data registry for breastfeeding and associated health outcomes could be instrumental for future research.

Despite limited evidence, potential benefits of breastfeeding on the mental health outcomes of both mother and child are apparent due to other known benefits. More efforts should be made that policies should promote and support breastfeeding practices, incorporating robust postpartum mental health screenings and targeted assistance programs for mothers.

## Conclusions

There is a lack of consistent evidence to support a relationship between breastfeeding and mental health outcomes in mothers and children. Some studies have found statistically significant associations between breastfeeding and mental health, while others have found no such associations. The quality and methods of these studies are inconsistent, making it difficult to draw conclusions about the relationship between breastfeeding and mental health. Further research is necessary to fully understand any potential associations and potential underlying mechanisms. While breastfeeding may have various benefits for both mothers and children, more research is needed to determine whether it can protect against the development of mental health disorders in both parties.

### Supplementary Information

**Additional file 1: Box S1.** Search strategies. **Table S1.** Newcastle Ottawa Scale (NOS) scoring for the cohort studies. **Table S2.** Newcastle Ottawa Scale (NOS) scoring for the case-control studies. **Table S3.** Newcastle Ottawa Scale (NOS) scoring for cross-sectional studies.

## Data Availability

The data that support the findings of this study are available from the corresponding author, DM, upon reasonable request.

## Abbreviations

AAP: American Academy of Pediatrics
ADHD: Attention deficit hyperactivity disorders
adjOR: Adjusted odds ratio
ASD: Autism spectrum disorders
BDI: Beck’s Depression Inventory
BPD: Borderline personality disorder
CBCL: Child Behaviour Check List
DSM: Diagnostic and Statistical Manual of Mental Disorders
ECPH: European Commission for Public Health
EPDS: Edinburgh Postnatal Depression Scale
GRADE: Grading of Recommendations, Assessment and Evaluation
GUTS: Growing Up Today Study
ICD: International Classification of Diseases
K-SADS: Schedule for Affective Disorders and Schizophrenia for School-Age Children
LC-PUFAs: Long-chain polyunsaturated fatty acids
MeSH: Medical subject headings
MINI-KID: Mini International Neuropsychiatric Interview for Children and Adolescents
NOS: Newcastle-Ottawa Scale
NPQ-PSQ: Pre-/Postnatal Stress Questionnaire
OR: Odds ratio
PHQ-9: Patient Health Questionnaire-9
PRISMA: Preferred Reporting Items for Systematic Reviews and Meta-Analyses
RCADS: Revised Child Anxiety and Depression Scale
RCT: Randomised controlled trial
ROBINS: Risk Of Bias In Non-randomised Studies—of Interventions
UK: United Kingdom
WHO: World Health Organization
YSR: Youth Self Report
