# Supplementary material for: Association of breastfeeding with mental disorders in mother and child: a systematic review and meta-analysis
Source: BMC Med. 2023 Oct 16;21:393. doi: 10.1186/s12916-023-03071-7 (PMC10577970; doi:10.1186/s12916-023-03071-7)
Supplement: Supplementary file 1 — Additional file 1: Box S1. Search strategies. Table S1. Newcastle Ottawa Scale (NOS) scoring for the cohort studies. Table S2. Newcastle Ottawa Scale (NOS) scoring for the case-control studies. Table S3. Newcastle Ottawa Scale (NOS) scoring for cross-sectional studies. [file 12916_2023_3071_MOESM1_ESM.docx]

**Association of breastfeeding with mental disorders in mother and child: a systematic review and meta-analysis**

**Supplementary Material**

Polina Bugaeva ^1,^*, Inna Arkusha ^2,3,^*, Rinat Bikaev ^3,^*, Igor Kamenskiy ^4^, Aleksandra Pokrovskaya ^5^, Yasmin El-Taravi ^6^, Valeria Caso ^7^, Alla Avedisova ^2^, Derek K Chu ^8^, Jon Genuneit ^9,10^, Gabriel Torbahn ^11,12^, Timothy Nicholson ^13^, Dina Baimukhambetova ^14^, Aigun Mursalova ^14^, Anastasia Kolotilina ^14^, Svetlana Gadetskaya ^14^, Elena Kondrikova ^14^, Mikhail Zinchuk ^3^, Renat Akzhigitov ^3^, Robert J Boyle ^15^, Alla Guekht ^3^, Daniel Munblit ^16,17^

1. Charité – Universitätsmedizin Berlin, Einstein Center for Neurosciences, Berlin, Germany
2. V. Serbsky Federal Medical Research Center for Psychiatry and Narcology of the Ministry of Health of the Russian Federation, Moscow, Russia
3. Moscow Research and Clinical Centre for Neuropsychiatry, Moscow, Russia
4. Moscow City Clinical Hospital after V.M. Buyanov, Moscow, Russia
5. Dementia Research Institute UK, Department of Brain Sciences, Faculty of Medicine, Imperial College London, London, UK
6. Endocrinology Research Centre, Moscow, Russia
7. Stroke Unit, Santa Maria Della Misericordia Hospital, University of Perugia, Perugia, Italy
8. Division of Clinical Immunology & Allergy, Department of Medicine, and Department of Health Research Methods, Evidence & Impact, McMaster University, Hamilton, Canada
9. Pediatric Epidemiology, Department of Pediatrics, Medical Faculty, Leipzig University, Leipzig, Germany
10. German Center for Child and Youth Health, Leipzig, Germany
11. Department of Pediatrics, Paracelsus Medical University, Klinikum Nürnberg, Universitätsklinik der Paracelsus Medizinischen Privatuniversität Nürnberg, Nuremberg, Germany
12. Department of Pediatrics, Paracelsus Medical University, Salzburg, Austria
13. Institute of Psychiatry, Psychology and Neuroscience, King’s College London, London, UK
14. Department of Paediatrics and Paediatric Infectious Diseases, Institute of Child’s Health, I.M. Sechenov First Moscow State Medical University, Sechenov University, Moscow, Russia
15. National Heart and Lung Institute, Imperial College London, London, UK
16. Care for Long Term Conditions Division, Florence Nightingale Faculty of Nursing, Midwifery and Palliative Care, King's College London, London, United Kingdom
17. I.M. Sechenov First Moscow State Medical University, Sechenov University, Moscow, Russia

**Table of contents**

[**Box S1. Search strategies** 3](#_Toc144819913)

[**Supplementary Table 1: Newcastle Ottawa Scale (NOS) scoring for the cohort studies** 7](#_Toc144819914)

[**Supplementary Table 2: Newcastle Ottawa Scale (NOS) scoring for the case-control studies** 9](#_Toc144819915)

[**Supplementary Table 3: Newcastle Ottawa Scale (NOS) scoring for cross-sectional studies** 10](#_Toc144819916)

| **Box S1. Search strategies**  **Database: Embase Classic+Embase (via Ovid)**  1 (Breastfeeding or Breast Feeding or Lactation or Human Milk or Breast Milk or Breast-fe* or Breast fe* or Breastfe* or bottle feeding or bottle fed or bottlefed or bottle feed or Infant Formula or Artificial formula or Breast milk substitute or Formula milk or Formula feed* or formula fed or weaning or wean*).ab,kw,ti.  2 exp breast milk/  3 exp breast feeding/  4 exp artificial milk/  5 exp infant nutrition/  6 exp weaning/  7 1 or 2 or 3 or 4 or 5 or 6  8 exp depression/  9 exp bipolar disorder/  10 exp anxiety disorder/  11 exp phobia/  12 exp panic/  13 exp obsessive compulsive disorder/  14 exp separation anxiety/  15 exp personality disorder/  16 exp neurosis/  17 exp anorexia nervosa/ or exp feeding disorder/  18 exp eating disorder/  19 exp bulimia/  20 exp binge eating disorder/  21 exp posttraumatic stress disorder/  22 exp gender dysphoria/ or exp dysphoria/  23 exp dissociative disorder/  24 exp autism/  25 exp schizophrenia/  26 exp mental disease/  27 exp emotional attachment/  28 exp suicide/  29 exp alcoholism  30 (depression or depress* disorder or depress* disease).ab,kw,ti.  31 (depressive adj2 disorder).ab,kw,ti.  32 (bipolar disorder or bipolar illness or bipolar illness or manic depressive disease or manic depressive disorder).ab,kw,ti.  33 (anxiety or anxiety disorder*).ab,kw,ti.  34 (phobia* or phobic fear* or phobic disorder*).ab,kw,ti.  35 (panic or panic disorder).ab,kw,ti.  36 (obsessive compulsive disorder or OCD or obsessive syndrome or obsess* neurosis or compuls* neurosis).ab,kw,ti.  37 (separation anxiety disorder or separation anxiety).ab,kw,ti.  38 "personality disorder*".ab,kw,ti.  39 (neuroticism or neurosis or neurotic disease or neurotic disorder).ab,kw,ti.  40 (anorexia nervosa or anorexia or feeding disorder or eating disorder or bulimia or binge eating disorder).ab,kw,ti.  41 (post-traumatic stress disorder or posttraumatic stress disorder or posttraumatic syndrome or PTSD).ab,kw,ti.  42 (dysphoria or dysforia).ab,kw,ti.  43 "dissociative disorder*".ab,kw,ti.  44 autism.ab,kw,ti.  45 schizophrenia.ab,kw,ti.  46 (emotional attachment or psychological attachment or mental attachment).ab,kw,ti.  47 (suicide or suicid* behavior).ab,kw,ti.  48 (alcoholism or alcohol addiction or alcohol dependenc* or ethanol dependenc* or dipsomania).ab,kw,ti.  49 8 or 9 or 10 or 11 or 12 or 13 or 14 or 15 or 16 or 17 or 18 or 19 or 20 or 21 or 22 or 23 or 24 or 25 or 26 or 27 or 28 or 29 or 30 or 31 or 32 or 33 or 34 or 35 or 36 or 37 or 38 or 39 or 40 or 41 or 42 or 43 or 44 or 45 or 46 or 47 or 48  50 7 and 49  51 limit 50 to human  **Database: Ovid MEDLINE(R)**  1 (Breastfeeding or Breast Feeding or Lactation or Human Milk or Breast Milk or Breast-fe* or Breast fe* or Breastfe* or bottle feeding or bottle fed or bottlefed or bottle feed or Infant Formula or Artificial formula or Breast milk substitute or Formula milk or Formula feed* or formula fed or weaning or wean*).ab,kf,ti.  2 exp Milk, Human/  3 exp Breast Feeding/  4 exp Infant Food/  5 exp Infant Formula/  6 exp Weaning/  7 1 or 2 or 3 or 4 or 5 or 6  8 exp Depression/  9 exp Bipolar Disorder/  10 exp Anxiety Disorders/  11 exp Phobic Disorders/  12 exp Panic Disorder/ or exp Panic/  13 exp Obsessive-Compulsive Disorder/  14 exp Anxiety, Separation/  15 exp Personality Disorders/  16 exp Neurotic Disorders/  17 exp Anorexia Nervosa/  18 exp "Feeding and Eating Disorders"/  19 exp Bulimia Nervosa/ or exp Bulimia/  20 exp Binge-Eating Disorder/  21 exp Stress Disorders, Post-Traumatic/  22 exp Gender Dysphoria/  23 exp Dissociative Disorders/  24 exp Autistic Disorder/  25 exp Schizophrenia/  26 exp Mental Disorders/  27 exp Suicide/  28 exp Alcoholism/  29 (depression or depress* disorder or depress* disease).ab,kf,ti.  30 (depressive adj2 disorder).ab,kf,ti.  31 (bipolar disorder or bipolar illness or bipolar illness or manic depressive disease or manic depressive disorder).ab,kf,ti.  32 (anxiety or anxiety disorder*).ab,kf,ti.  33 (phobia* or phobic fear* or phobic disorder*).ab,kf,ti.  34 (panic or panic disorder).ab,kf,ti.  35 (obsessive compulsive disorder or OCD or obsessive syndrome or obsess* neurosis or compuls* neurosis).ab,kf,ti.  36 (separation anxiety disorder or separation anxiety).ab,kf,ti.  37 "personality disorder*".ab,kf,ti.  38 (neuroticism or neurosis or neurotic disease or neurotic disorder).ab,kf,ti.  39 (anorexia nervosa or anorexia or feeding disorder or eating disorder or bulimia or binge eating disorder).ab,kf,ti.  40 (post-traumatic stress disorder or posttraumatic stress disorder or posttraumatic syndrome or PTSD).ab,kf,ti.  41 (dysphoria or dysforia).ab,kf,ti.  42 "dissociative disorder*".ab,kf,ti.  43 autism.ab,kf,ti.  44 schizophrenia.ab,kf,ti.  45 (emotional attachment or psychological attachment or mental attachment).ab,kf,ti.  46 (suicide or suicid* behavior).ab,kf,ti.  47 (alcoholism or alcohol addiction or alcohol dependenc* or ethanol dependenc* or dipsomania).ab,kf,ti.  48 8 or 9 or 10 or 11 or 12 or 13 or 14 or 15 or 16 or 17 or 18 or 19 or 20 or 21 or 22 or 23 or 24 or 25 or 26 or 27 or 28 or 29 or 30 or 31 or 32 or 33 or 34 or 35 or 36 or 37 or 38 or 39 or 40 or 41 or 42 or 43 or 44 or 45 or 46 or 47  49 7 and 48  50 limit 49 to humans |
| --- |

**Table S1: Newcastle Ottawa Scale (NOS) scoring for the cohort studies**

| **STUDY ID** | **Author, year** | **Representativenes of the exposed**  **cohort** | **Selection of non-exposed cohort** | **Ascertainement of exposure** | **Demonstration that outcome of interest was not present at start of study** | **Comparability of cohorts for THE MOST IMPORTANT FACTOR** | **Comparability of cohorts for THE ADDITIONAL FACTOR** | **Assessment of outcome** | **Was follow-up long enough for outcomes to occur** | **Adequacy of follow-up of cohorts** | **Total** |
| --- | --- | --- | --- | --- | --- | --- | --- | --- | --- | --- | --- |
| 10.1089/bfm.2011.0136 | Hayatbakhsh, 2012 | 1 | 1 | 0 | 1 | 1 | 1 | 0 | 1 | 0 | 6 |
| https://doi.org/10.1111/j.1600-0447.2005.00548.x | Sorensen, 2005 | 1 | 1 | 1 | 1 | 1 | 1 | 1 | 1 | 1 | 8 |
| 10.1002/eat.22165 | Iron-Segev, 2013 | 0 | 1 | 0 | 1 | 1 | 0 | 0 | 1 | 0 | 4 |
| 10.1089/bfm.2018.0151 | Huang, 2018 | 1 | 1 | 1 | 0 | 1 | 1 | 0 | 0 | 1 | 6 |
| [10.1016/j.jpeds.2009.10.020](https://doi.org/10.1016/j.jpeds.2009.10.020) | Oddy, 2010 | 1 | 1 | 1 | 1 | 1 | 1 | 1 | 1 | 0 | 8 |
| https://doi.org/10.1016/j.jad.2018.12.081 | Park, 2018 | 1 | 1 | 0 | 0 | 1 | 1 | 0 | 0 | 1 | 5 |
| DOI 10.1002/da.22109 | Zhong, 2013 | 1 | 1 | 0 | 1 | 1 | 1 | 1 | 0 | 1 | 6 |
| doi: 10.1136/archdischild-2013-304250 | Kwok, 2013 | 1 | 1 | 0 | 1 | 1 | 1 | 1 | 1 | 0 | 7 |
| doi.org/10.1016/j.jad.2016.05.055 | de Mola, 2016 | 1 | 1 | 0 | 1 | 1 | 0 | 1 | 1 | 0 | 4 |
| 10.1017/s0954579498001722 | Allen, 1998 | 1 | 1 | 0 | 1 | 1 | 0 | 1 | 1 | 0 | 5 |
| http://dx.doi.org/10.1089/bfm.2013.0142 | Xu, 2014 | 1 | 1 | 1 | 0 | 1 | 1 | 1 | 1 | 1 | 7 |
| http://dx.doi.org/10.1007/s00737-013-0348-9 | Hahn-Holbrook, 2013 | 1 | 1 | 1 | 0 | 1 | 1 | 1 | 1 | 1 | 7 |
| 10.1192/bjp.170.4.334 | McCreadi, 1997 | 1 | 1 | 0 | 1 | 1 | 1 | 1 | 0 | 1 | 7 |

**Table S2: Newcastle Ottawa Scale (NOS) scoring for the case-control studies**

| **STUDY ID** | **AUTHOR, year** | **Is the case definition adequate?** | **Representativeness of the cases** | **Selection of controls** | **Definition of controls** | **Comparability of cases and controls for THE MOST IMPORTANT FACTOR** | **Comparability of cases and controls for THE ADDITIONAL FACTOR** | **Ascertainement of exposure** | **Same method of ascertainement for cases and controls** | **Non-response rate** | **Total** |
| --- | --- | --- | --- | --- | --- | --- | --- | --- | --- | --- | --- |
| http://dx.doi.org/10.1521/pedi_2014_28_160 | Schwarze, 2014 | 1 | 0 | 0 | 1 | 1 | 1 | 1 | 1 | 0 | 7 |
| 10.4102/sajpsychiatry.v13i2.30 | Hartog, 2007 | 1 | 0 | 0 | 1 | 1 | 1 | 0 | 1 | 1 | 6 |
| 10.1016/s0920-9964(99)00102-4 | Sasaki, 2000 | 1 | 0 | 1 | 1 | 1 | 0 | 0 | 1 | 0 | 5 |
| 10.1159/000067960 | Amore, 2003 | 1 | 1 | 0 | 0 | 1 | 1 | 0 | 1 | 1 | 6 |
| http://dx.doi.org/10.1089/bfm.2018.0013 | Orengul, 2018 | 1 | 0 | 1 | 1 | 1 | 1 | 1 | 1 | 1 | 8 |
| http://dx.doi.org/10.1192/bjp.177.3.218 | Leask, 2000 | 1 | 1 | 1 | 1 | 1 | 1 | 1 | 1 | 0 | 8 |

**Table S3: Newcastle Ottawa Scale (NOS) scoring for cross-sectional studies**

| **STUDY ID** | **AUTHOR, year** | **Representativeness of the sample** | **Sample size** | **Non-respondents** | **Ascertainment of the exposure** | **Comparability:**  **The study controls for the most important factor** | **Comparability:**  **The study control for any additional factor** | **Outcome** | **Statistical test** | **Total** |
| --- | --- | --- | --- | --- | --- | --- | --- | --- | --- | --- |
| https://doi.org/10.1007/s00406-022-01477-7 | Liu, 2023 | 1 | 1 | 0 | 0 | 1 | 1 | 1 | 1 | 6 |
